# Effects of the application of different improved materials on reclaimed soil structure and maize yield of Hollow Village in Loess Area

**DOI:** 10.1038/s41598-022-10898-2

**Published:** 2022-05-06

**Authors:** Zhe Liu, Yang Zhang, Zenghui Sun, Yingying Sun, Huanyuan Wang, Ruiqing Zhang

**Affiliations:** 1 Shaanxi Provincial Land Engineering Construction Group Co., Ltd., Xi’an, 710075 China; 2 Key Laboratory of Degraded and Unused Land Consolidation Engineering, The Ministry of Natural Resources, Xi’an, 710021 China; 3 Institute of Land Engineering and Technology, Shaanxi Provincial Land Engineering Construction Group Co., Ltd., Xi’an, 710021 China; 4 Shaanxi Provincial Land Consolidation Engineering Technology Research Center, Xi’an, 710075 China; 5 School of Human Settlements and Civil Engineering, Xi’an Jiaotong University, Xi’an, 710049 China

**Keywords:** Agroecology, Ecology, Environmental sciences

## Abstract

In order to solve the soil problem of poor structure and low fertility after the abandoned homestead reclamation of Hollow Village in Loess Area and to improve the quality of the reclaimed soil in Hollow Village, a five-year field experiment was conducted here. In this experiment, the following seven treatments were applied: no modified material (CK), maturing agent (TM), fly ash (TF), organic fertilizer (TO), maturing agent + fly ash (TMF), maturing agent + organic fertilizer (TMO) and fly ash + organic fertilizer (TFO), and the effects of different improved materials on soil properties and crop yield were studied. The results showed that: soil organic matter (SOM) and total nitrogen contents increased significantly after the application of different improved materials, which promoted the cementation and aggregation of water-stable microaggregates (< 0.25 mm), and the water-stable macroaggregates showed an increasing trend. In the 0–0.15 m soil layer, the proportion of water-stable macroaggregates under TM, TF, TO, TMF, TMO, and TFO treatment increased by 328.2%, 130.0%, 87.8%, 81.1%, 36.7%, and 12.2% compared with CK, respectively. Meanwhile, TF, TO, TMF, TMO, TFO treatments significantly increased the mean weight diameter (MWD) and geometric mean diameter (GMD) values, reduced soil bulk density, the stable aggregate index (*E*_*LT*_) and fractal dimension (*D*) values (*P* < 0.05), and the stability of soil structure and the capacity of soil moisture retention has been significantly improved. The SOM content had a significant positive correlation with MWD, GMD, and > 2 mm water-stable aggregates and a significant negative correlation with the *E*_*LT*_, *D*, and water-stable microaggregates. In particular, the organic–inorganic coupling treatment of TFO showed the highest SOM content, soil moisture content, water-stable macroaggregates and maize yield, which was the most appropriate amendment for improving the reclaimed soil structure and fertility of Hollow Village in Loess Area.

## Introduction

At present, the loess plateau region is facing the problems of fragile ecological conditions, decreasing arable land area, lack of arable land reserve resources, and intensifying human-land conflicts. With the rapid promotion and implementation of urbanization and industrialization, a large number of rural laborers migrate to cities, resulting in the continuous deterioration of abandoned and hollowed rural areas^1,2^. Therefore, a great deal of abandoned housing resources is left idle and wasted^3^. Moreover, the rural housing land construction generally shows an undesirable trend of "building the new but not demolishing the old", occupying high-quality arable land to expand to the periphery, resulting in the destruction and occupation of a large number of arable land resources^4,5^. Rural hollowing seriously threatens arable land resources and regional food security, and has become a major bottleneck limiting the construction of villages and the coordinated development of urban and rural areas^4–6^. In view of the above problems, combined with the rising contradiction between human and land in the loess plateau region and the increasing local demand for reclaiming the abandoned housing land, it is necessary to promote comprehensive land improvement in hollow villages and carry out reclamation of abandoned housing land for implementing "requisition-compensation balance" of arable land and alleviating the regional contradiction between human and land^6–8^. However, hollow village reclaimed soil mainly comes from the old wall soil on the abandoned housing land, which is mostly raw soil that has not been cultivated for years due to the combined influence of natural and human factors, and has lost its natural functions and properties. Its physical structure and soil fertility are seriously damaged, which limits the land productivity and sustainable development of new arable land in hollow villages. It is necessary to improve the structural properties and fertility features of the reclaimed soil on the housing land and to improve the productivity of the reclaimed soil^9,10^.

The number and distribution ratio of soil aggregates are not only important indicators of soil erosion, hardening, compaction and other structural conditions, but also play a key role in supplying and storing soil nutrients, regulating soil water holding capacity and maintaining land productivity, and they are key indicators that can well evaluate the changes in soil fertility feature and environmental quality1^11–13^. Compared with non-water-stable aggregates, water-stable aggregates play a more important role in maintaining soil structural stability and erosion resistance, and retaining soil nutrients^14,15^. It was found that the increase of organic matter content is directly related to the increase of the number and stability of water-stable aggregates, and the application of different improved materials can effectively increase the organic matter content of the soil, promote the cemented agglomeration and structural stability of soil aggregates, and improve the fertility and erosion resistance of reclaimed soil^16,17^. Fly ash is the powdery solid residue discharged from coal-fired power plant after the combustion of pulverized coal, which contained abundant clay particles and oxides such as Al_2_O_3_ and Fe_2_O_3_, it could significantly improve mutual adsorption and aggregation between soil particles and enhance the soil water and fertility retention^18,19^. The maturing agent of ferrous sulfate can improve soil structure and reduce the pH value of soil while loosening the soil, it can also be used as fertilizer, which plays an important role in the absorption of plants^20^. Organic fertilizers are rich in organic matter and various nutrients, which can effectively improve soil fertility and structure, increase soil productivity and maintain the sustainability of crop yield^21,22^. Therefore, it is of practical importance and necessity to enhance the structural stability and fertility feature of the reclaimed soil by applying different soil improved materials to mature and improve the reclaimed soil of hollow villages, so that the reclaimed soil can recover its original functions and properties. The current approaches to remediation of hollow villages are mainly focused on the assessment of remediation potential, evolution laws, regional macro-remediation models and regulation policies, which further affirm the significance of the remediation of abandoned housing land in hollow villages^23–25^. However, there are few studies on the effects of different improved materials on hollow village reclaimed soil, which seriously restrict the availability and sustainable development of hollow village reclaimed soil.

The reclaimed soil in hollow villages is taken as a key initiative to the regional supplementation to arable land resources; however, little research is conducted to compare and analyze the role of different improved materials in the structure and fertility improvement of reclaimed soil, thus it is difficult to distinguish the differences in influences of different improved materials on the number of aggregates, structural stability, nutrient content and crop yield of reclaimed soils. In order to make up for the lack of information about the effect of different improved materials on the hollow village reclaimed soil, the objectives of this paper were to evaluate : (1) the effects of different improved materials on soil nutrient content, aggregate distribution and stability, soil bulk density and soil moisture content; (2) the correlation between the soil organic matter and the water-stable aggregates distribution and stability indexes; and (3) the effects of different improved materials on maize yield in hollow village reclaimed soil. The research results will provide a theoretical basis for selecting appropriate amendment materials to improve the structure and quality of reclaimed soil in the hollow village.

## Results and analysis

### Effects of the application of different improved materials on properties of reclaimed soil

#### Soil organic matter (SOM) and total nitrogen (TN)

**Figure 1:**
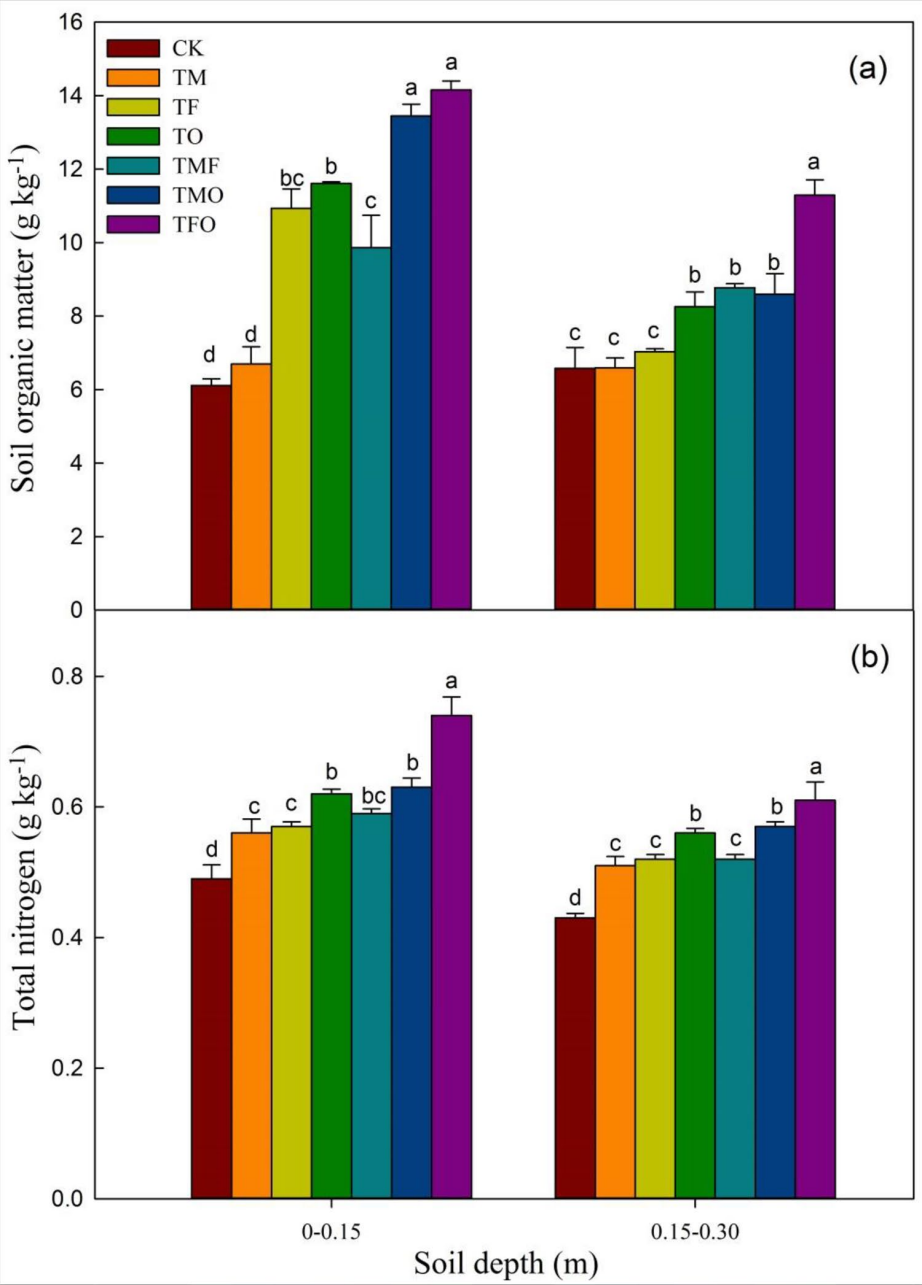
Effects of the application of different improved materials on SOM and TN CK: no improved material; TM: maturing agent (ferrous sulfate); TF: fly ash; TO: organic fertilize; TMF: maturing agent + fly ash, TMO: maturing agent + organic fertilizer; TFO: fly ash + organic fertilizer; SOM, soil organic matter; TN, total nitrogen. Different lowercase letters represent significant differences among different improved material treatments in the same soil layer.

After the application of different improved materials, the SOM and TN contents in both 0–0.15 m and 0.15–0.30 m layers of the hollow village reclaimed soil showed an overall increasing trend (Fig. 1). In the 0–0.15 m layer, the organic matter content increased by 9.6%, 79.0%, 90.0%, 61.4%, 120.1%, and 131.7% respectively under TM, TF, TO, TMF, TMO and TFO treatments compared with CK treatment, indicating that different improved materials all played important roles in improving the organic matter content of reclaimed soil (Fig. 1a). The improvement of organic matter content in the 0–0.15 m layer of reclaimed soil by the treatments of different improved materials showed as follows: TFO > TMO > TO > TF > TMF > TM > CK, and TO, TMO and TFO treatments with organic fertilizer addition could significantly improve the organic matter content of the reclaimed soil (*P* < 0.05), among which TFO treatment was the most effective on improvement of the organic matter content. In the 0.15–0.30 m layer, the results of significance analysis showed that TO, TMF, TMO and TFO treatments all significantly increased the organic matter content (*P* < 0.05), while TM and TF treatments had no significant difference in improving the soil organic matter content, with TFO treatment having the most significant effect.

Compared with CK, the concentration of TN in the two soil layers had similar increasing trends to SOM after the application of different improved materials (Fig. 1b). In the 0–0.15 m layer, TM, TF, TO, TMF, TMO and TFO increased by 14.29%, 16.33%, 26.53%, 20.41%, 28.57%, and 51.02%, respectively. In the 0.15–0.30 m layer, the concentration of TN also showed an increasing trend in varying degrees.

#### Size distribution of water-stable aggregates

**Figure 2:**
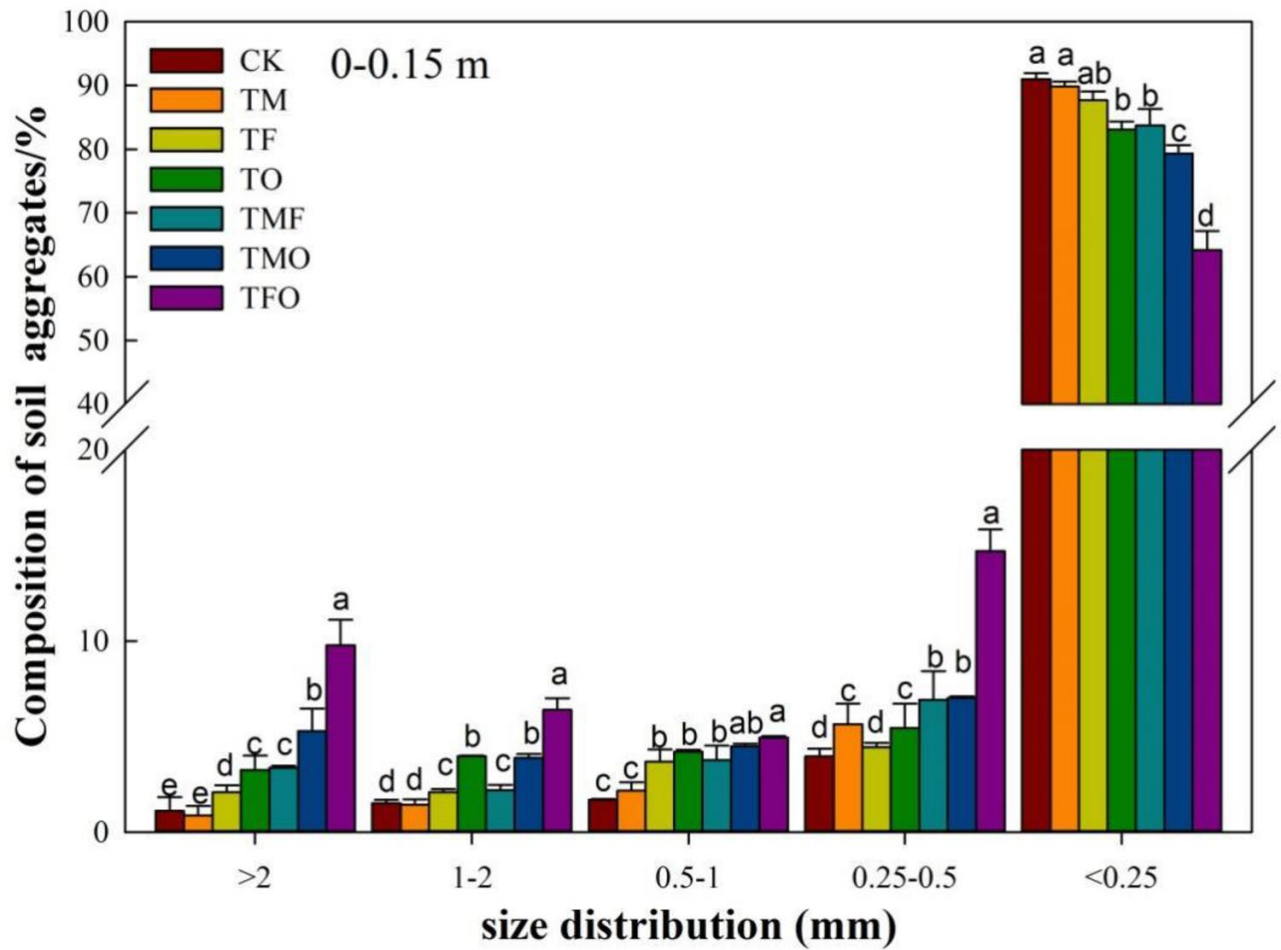
Percentage (%) of soil water-stable aggregates under the application of different improved materials at 0.15–0.30 m Depth. CK: no improved material; TM: maturing agent (ferrous sulfate); TF: fly ash; TO: organic fertilize; TMF: maturing agent + fly ash, TMO: maturing agent + organic fertilizer; TFO: fly ash + organic fertilizer. Different lowercase letters represent significant differences among different improved material treatments in the same particle-size aggregates.

**Figure 3:**
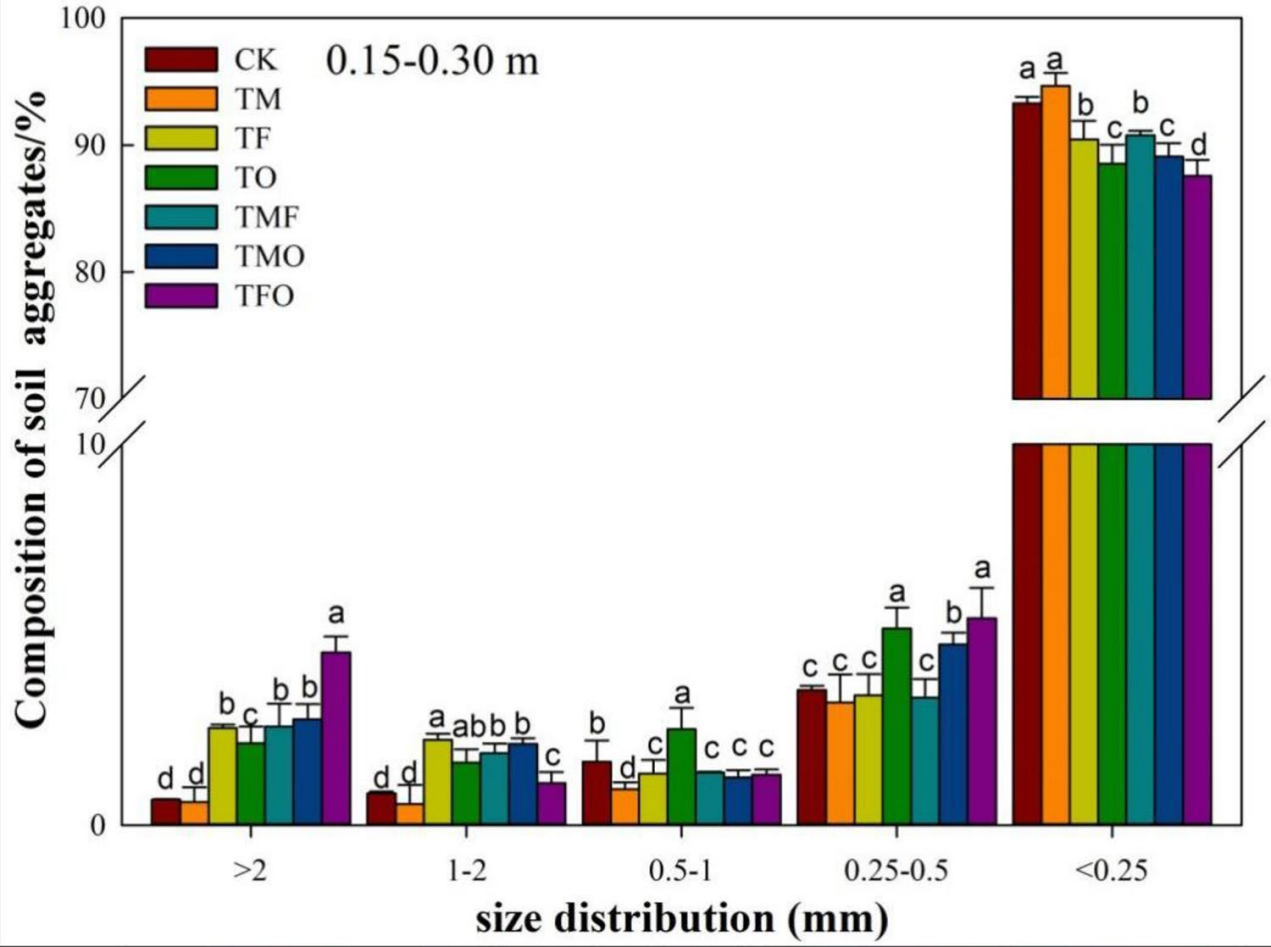
Percentage (%) of soil water-stable aggregates under the application of different improved materials at 0.15–0.30 m Layer. CK: no improved material; TM: maturing agent (ferrous sulfate); TF: fly ash; TO: organic fertilize; TMF: maturing agent + fly ash, TMO: maturing agent + organic fertilizer; TFO: fly ash + organic fertilizer. Different lowercase letters represent significant differences among different improved material treatments in the same particle-size aggregates.

Water-stable aggregates are important indicators for evaluating the structural stability and erosion resistance of soil, and their quantity and distribution can well reflect the changes in soil structure and quality^26^. Compared with CK, significant changes in the distribution of water-stable aggregates were shown in the reclaimed soils in the 0–0.15 m and 0.15–0.30 m layers after the application of different improved materials (Figs. 2 and 3) (*P* < 0.05). In the 0–0.15 m layer, after the application of different improved materials in hollow village reclaimed soil, the proportion of water-stable macroaggregates (particle size > 0.25 mm) showed an overall increasing trend, and the water-stable microaggregates content (particle size < 0.25 mm) showed a decreasing trend. In particular, it showed that except for the treatment of maturing agent (TM), the proportion of water-stable aggregates (particle size > 2 mm, 1–2 mm, and 0.5–1 mm) were significantly increased under the TF, TO, TMF, TMO and TFO compared with CK, especially that of particle size > 2 mm (Fig. 2). The proportion of > 2 mm water-stable aggregates was increased by 88.1%, 194.5%, 203.7%, 376.2%, and 781.7% respectively under TF, TO, TMF, TMO and TFO compared with CK. The proportion of water-stable macroaggregates under different treatments showed as follows: TFO (35.8%) > TMO (20.7%) > TO (16.9%) > TMF (16.3%) > TF (12.3%) > TM (10.1%) > CK (9.0%), and the water-stable macroaggregates were increased by 328.2%, 130.0%, 87.8%, 81.1%, 36.7%, and 12.2% respectively compared with CK, with the maximum increase of 328.2%. In general, all six different amendment material treatments increased the proportion of water-stable macroaggregates in reclaimed soil and promoted the aggregation and cementation of water-stable microaggregates (< 0.25 mm) to water-stable macroaggregates (> 0.25 mm). And the TFO showed the best effect on the increase of water-stable macroaggregates, followed by TMO, TO, and TMF, while TF and TM treatments showed little effect.

In the 0.15–0.30 m layer, the change of water-stable aggregates showed a similar trend to that in the 0–0.15 m layer compared with CK treatment. TF, TO, TMF, TMO and TFO treatments all significantly increased the proportion of > 2 mm and 1–2 mm water-stable aggregates, and decreased the proportion of water-stable microaggregates (*P* < 0.05) (Fig. 3). In particular, TF, TO, TMF, TMO and TFO treatments increased the proportion of > 2 mm water-stable aggregates by 130.3%, 94.5%, 133.9%, 151.4%, and 309.2% respectively compared with CK, of which TFO treatment showed the most significant effect on the increase of the proportion of water-stable macroaggregates. Compared with the 0–0.15 m layer, the proportion of water-stable macroaggregates in the 0.15–0.30 m layer showed a gradual decrease with the increase of soil depth.

#### Water-stable aggregates structure stability

The mean weight diameter (MWD), geometric mean diameter (GMD), unstable aggregate index (*E*_*LT*_), and fractal dimension (*D*) are important indicators reflecting the structural geometry and stability of soil aggregates, and it has been indicated in this research that the higher the MWD and GMD and the smaller the *E*_*LT*_ and *D*, the better the structural stability of the aggregates and the soil structure^27,28^. Compared with CK treatment, the MWD and GMD showed a trend of significant increase while the *D* and *E*_*LT*_ showed a trend of significant decrease (*P* < 0.05) under TF, TO, TMF, TMO and TFO treatments after the application of different improved materials, and TM treatment had no significant effect on the indicators of aggregate stability (Table 1). In the 0–0.15 m layer, the MWD is increased by 6.19%, 27.66%, 22.16%, 49.71% and 125.96% and the GMD is increased by 4.09%, 12.46%, 9.34%, 19.82% and 49.15% respectively under TF, TO, TMF, TMO and TFO treatments compared with CK treatment, while the *E*_*LT*_ is decreased by 1.35% to 29.5%, and the *D* is decreased by 0.76% to 4.35% respectively compared with CK treatment. TF, TO, TMF, TMO and TFO treatments all improved the aggregation capacity of aggregates to different degrees and enhanced the structural stability and erosion resistance of the reclaimed soil, with TFO treatment having the best effect on improving the structural stability of aggregates. In the 0.15–0.30 m layer, the MWD, GMD, *D*, and *E*_*LT*_ also show significant increase under TF, TO, TMF, TMO and TFO treatments compared with CK treatment (*P* < 0.05). It can be seen from the data of aggregate stability indicators that the structural stability of water-stable aggregates in the 0.15–0.30 m layer shows a decreasing trend compared with the 0–0.15 m layer, which may be related to the higher organic content in the 0–0.15 m layer.

**Table 1:** Effects of the application of different improved materials on water-stable aggregate stability indexes. GMD, geometric mean diameter; MWD, mean weight diameter; *E*_*LT*_, unstable aggregate index; *D*, fractal dimension. Different lowercase letters represent significant differences among different improved material treatments in the same aggregate stability index.

Soil layer	Treatments	MWD (mm)	GMD (mm)	*E*_*LT*_/%	*D*
0–0.15 m	CK	0.32 ± 0.03d	0.28 ± 0.01c	91.01 ± 0.42a	2.97 ± 0.01a
	TM	0.32 ± 0.02d	0.28 ± 0.01c	89.87 ± 0.15ab	2.99 ± 0.00a
	TF	0.38 ± 0.02c	0.29 ± 0.01bc	87.69 ± 1.38b	2.97 ± 0.01a
	TO	0.45 ± 0.02c	0.31 ± 0.01b	83.08 ± 1.30b	2.95 ± 0.01b
	TMF	0.43 ± 0.01c	0.31 ± 0.01b	83.72 ± 2.62b	2.95 ± 0.01b
	TMO	0.53 ± 0.05b	0.34 ± 0.01b	79.31 ± 1.35b	2.92 ± 0.02b
	TFO	0.80 ± 0.06a	0.42 ± 0.02a	64.17 ± 3.02c	2.84 ± 0.02c
0.15–0.30 m	CK	0.30 ± 0.01c	0.27 ± 0.00c	93.28 ± 0.52a	2.99 ± 0.01a
	TM	0.29 ± 0.02c	0.26 ± 0.01c	94.66 ± 1.03a	2.99 ± 0.00a
	TF	0.39 ± 0.01b	0.29 ± 0.01b	90.43 ± 1.47b	2.96 ± 0.01b
	TO	0.37 ± 0.02b	0.29 ± 0.01b	88.50 ± 1.54bc	2.97 ± 0.01b
	TMF	0.39 ± 0.03b	0.29 ± 0.01b	90.77 ± 0.35b	2.96 ± 0.01b
	TMO	0.39 ± 0.01b	0.29 ± 0.00b	89.07 ± 1.07b	2.96 ± 0.01b
	TFO	0.48 ± 0.02a	0.30 ± 0.01a	87.58 ± 1.06c	2.93 ± 0.01c

#### Soil bulk density and soil moisture content

**Figure 4:**
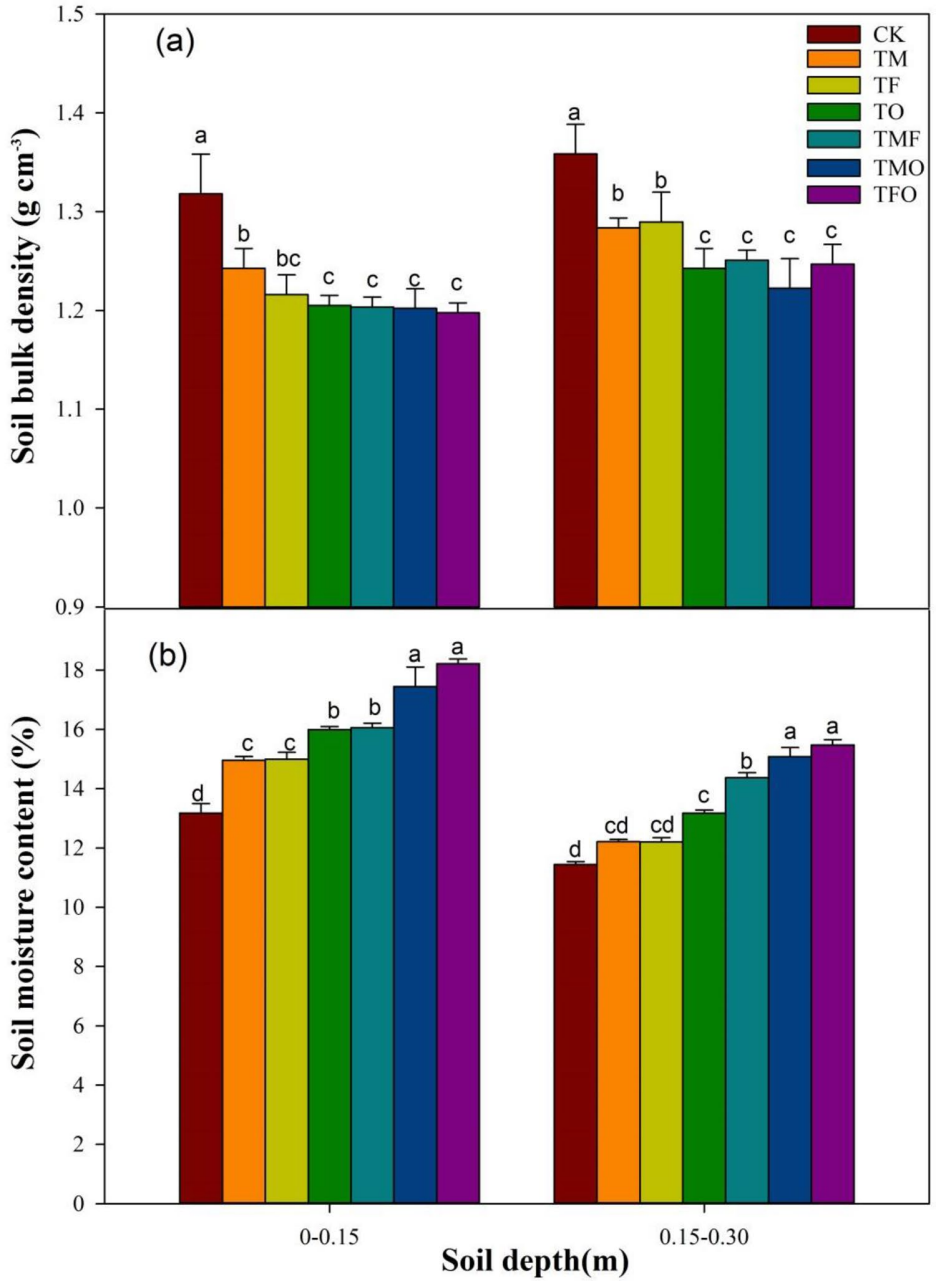
Effects of the application of different improved materials on BD and SMC. CK: no improved material; TM: maturing agent (ferrous sulfate); TF: fly ash; TO: organic fertilize; TMF: maturing agent + fly ash, TMO: maturing agent + organic fertilizer; TFO: fly ash + organic fertilizer; BD, soil bulk density; SMC, soil moisture content. Different lowercase letters represent significant differences among different improved material treatments in the same soil layer.

Soil bulk density (BD) is one of the important indicators reflecting soil quality, and the BD at the 0–0.15 m and 0.15–0.30 m layers of the reclaimed soil decreased significantly after the application of different improved materials (*P* < 0.05) (Fig. 4a). In the 0–0.15 m layer, the BD, under TM, TF, TO, TMF, TMO and TFO treatments, was decreased by 5.71%, 7.74%, 8.57%, 8.69%, 8.79% and 9.13% respectively compared with CK, which indicated that the application of different improved materials all have a negative effect on the BD to a certain degree. However, the loosening effect on the reclaimed soil was different due to the different characteristics of the improved materials, and the BD of reclaimed soil under different treatments showed as follows: TFO > TMO > TO > TF > TMF > TM > CK. The combination of organic–inorganic improved materials can effectively reduce the BD of reclaimed soil, and the BD under TFO treatment was the smallest, 1.19 g cm^−3^. In the 0.15–0.30 m layer, through variance analysis, the effect of different improved materials on the BD showed a similar decreasing trend to that in the 0–0.15 m layer.

The soil moisture content (SMC) of the reclaimed soil in the 0–0.15 m and 0.15–0.30 m layers increased significantly after the application of different improved materials (*P* < 0.05), and the variation of SMC in the two soil layers under different treatments was basically similar, showing as follows: TFO > TMO > TMF > TO > TF≈TM > CK (Fig. 4b). In the 0–0.15 m soil layer, the SMC under TM, TF, TO, TMF, TMO and TFO treatments was increased by13.5%, 13.8%, 21.4%, 21.9%, 32.4% and 38.3% respectively compared with CK. The TMO and TFO showed the most significant positive effect on the SMC of reclaimed soil, and the mass water content was 17.4% and 18.2% respectively. In conclusion, compared with CK, these improved materials increased the SOM content and porosity, promoted the formation and stability of aggregates, and increased the retention and transmission of water, which was helpful to maintain more water. Among them, the coupling treatment of organic and inorganic improved materials can hold more soil moisture, and the most significant increase was observed under TFO and TMO.

### Correlation analysis between soil organic matter and water-stable aggregates parameters

To further explore the correlation between the parameters of the reclaimed soil after the application of six different improved materials, a regression analysis was conducted in this paper on the correlation between the parameters of organic matter and water-stable aggregates with different particle sizes. From Table 2, it could be seen that the organic matter content had a highly significant positive correlation with MWD, GMD and > 2 mm water-stable aggregates content and a highly significant negative correlation with *E*_*LT*_, *D* and water-stable microaggregates content (< 0.25 mm), indicating that soil organic matter was an important factor affecting the formation of water-stable aggregates and their structural stability., and higher organic matter content would promote the formation of macro water-stable aggregates and improve the structural stability of soil. The water-stable aggregates (particle size > 2 mm, 1–2 mm, and 0.5–1 mm) content had a significant positive correlation with MWD and GMD values and a highly significant negative correlation with *E*_*LT*_ and *D* values; water-stable microaggregates (< 0.25 mm) had a highly significant negative correlation with MWD and GMD values and a significant positive correlation with *D*, indicating that the increase of water-stable aggregates with larger particle size helped to promote the structural stability of soil aggregates. In summary, it showed that TM, TF, TO, TMF, TMO and TFO treatments of improved materials can effectively promote the formation of macro water-stable aggregates and improve their structural stability while promoting the increase of organic matter content in the hollow village reclaimed soil. In particular, TFO treatment was more beneficial to improve the structural properties of hollow village reclaimed soil, enrich the soil fertility, and enhance the erosion resistance.

**Table 2:** Correlation analysis between SOM and water-stable aggregates parameters. SOM, soil organic matter; GMD, geometric mean diameter; MWD, mean weight diameter; *E*_*LT*_, unstable aggregate index; *D*, fractal dimension. * means significant correlation at 0.05 level; ** means highly significant correlation at 0.01 level.

Index	SOM (g kg^−1^)	WMD (mm)	GMD (mm)	*E*_*LT*_ (%)	*D*	Size (mm)				
						> 2	1–2	0.5–1	0.25–0.5	< 0.25
SOM	1									
WMD	0.7177**	1								
GMD	0.6960**	0.9798**	1							
*E*_*LT*_	−0.6948**	−0.9364**	−0.9814**	1						
*D*	−0.7003**	−0.9926**	−0.9540**	0.9001**	1					
> 2	0.7316**	0.9846**	0.9457**	−0.8948**	−0.9935**	1				
1–2	0.5949*	0.8140**	0.8835**	−0.8881**	−0.7639**	0.7596**	1			
0.5–1	0.5450	0.8522*	0.8980*	−0.9256**	−0.8190**	0.8080**	0.6602**	1		
0.25–0.5	0.5521	0.4727	0.5708	−0.6744**	−0.4167	0.4234	0.7138*	0.5190	1	
< 0.25	−0.6948**	−0.9364**	−0.9814**	1.0000	0.9001**	−0.8948**	−0.8881**	0.6744**	−0.9256**	1

### Effects of application of different improved materials on maize yield

Different improved materials showed a different effect on maize yield (Table 3). In particular, the effect of different treatments on maize yield showed as follows: TFO > TMO > TO > TMF > TF > TM > CK, and different improved materials all significantly increased maize yield compared with CK (*P* < 0.05). The average kernels per ear and 100-kernel weight under different treatments showed a similar increasing trend to the maize yield, highest under TFO, following by TMO and TO. Compared with CK, the 100-grain weight increased by 2.0%, 3.9%, 8.1%, 4.8%, 4.9% and 12.5% respectively under TM, TF, TO, TMF, TMO and TFO treatments, and the maize yield increased by 10.1%, 18.2%, 34.1%, 24.9%, 38.8% and 53.4%, respectively. The maize yield under TFO was the highest, up to 11,558.79 kg ha^−1^. In summary, it showed that organic improved materials had better effect on improving the maize yield than inorganic improved materials. The organic–inorganic coupling treatment of TFO and TMO had the best effect on improving the 100-grain weight and maize yield of the reclaimed soil. The possible reason was that the combination of organic and inorganic constituents can effectively increase the soil organic matter and total nitrogen contents, promote the formation and cementation of aggregates, increase the retention and transmission of water and improve the structural stability of hollow village reclaimed soil, which were confirmed by the results of the previous effects on SOM, total nitrogen, soil moisture content, aggregate proportion and aggregates structural stability index.

**Table 3:** Maize yield under different improved material treatments. CK: no modified material; TM: maturing agent (ferrous sulfate); TF: fly ash; TO: organic fertilize; TMF: maturing agent + fly ash, TMO: maturing agent + organic fertilizer; TFO: fly ash + organic fertilizer; BD, soil bulk density; SMC, soil moisture content. Different lowercase letters represent significant differences among different improved material treatments in the same indicator.

Treatments	Row number /ear	Kernels/row	Kernels/ear	100-kernel weight (g)	Theoretical yield (Kg ha^−1^)
CK	14.67 ± 1.15b	34.33 ± 1.53c	500.22 ± 25.73e	27.38 ± 0.38d	7532.48f.
TM	14.67 ± 0.58b	35.67 ± 0.58bc	516.44 ± 15.79de	27.93 ± 0.25 cd	8293.37e
TF	15.00 ± 1.00ab	36.67 ± 1.00bc	544.67 ± 10.67 cd	28.44 ± 0.98 cd	8906.10de
TO	15.33 ± 1.15ab	38.67 ± 1.15a	593.78 ± 16.80b	29.59 ± 0.80b	10,102.46bc
TMF	15.33 ± 1.15ab	37.33 ± 1.52ab	570.44 ± 8.46bc	28.69 ± 0.15bc	9411.57bcd
TMO	15.67 ± 0.58ab	38.67 ± 1.15a	606.22 ± 9.79b	28.73 ± 0.99bc	10,451.27b
TFO	16.67 ± 1.54a	39.33 ± 1.15a	652.44 ± 13.81a	30.81 ± 0.16a	11,558.79a

## Discussion

### The effects of the application of different improved materials on the water-stable aggregates and crop yield in reclaimed soil of hollow village

Soil organic matter, oxides and clay minerals are the main cementing substances for the formation and stability of soil aggregates, and play important roles in the content, size distribution, and structural stability of soil aggregates^29,30^. Improved materials such as organic fertilizer, maturing agent (ferrous sulfate) and fly ash can effectively improve soil organic matter content when applied alone or returned to the field after coupling. The cementing substances formed during conversion and decomposition contribute to the cementation and aggregation of soil aggregates and have significant effects on the size distribution and structural stability of the aggregates^12,31^. In this study, except for TM treatment, TF, TO, TMF, TMO and TFO all significantly increased the > 2 mm and 1–2 mm water-stable aggregates content and decreased water-stable microaggregates content (*P* < 0.05) compared with CK. In particular, the treatment of fly ash + organic fertilizer (TFO) had more positive effects on the water-stable macroaggregates content, which may be partially attributed to the higher SOM content under the TFO treatment. Our results are partially consistent with that of Singh et al*.* and Zhang et al*.*, who found that the combined application of organic and inorganic improved materials showed higher SOM content than the application of either amendment material alone, and therefore had a more positive effect on promoting the formation and preservation of macroaggregates^32,33^.

The Study by Blissett et al*.* showed that, as fly ash had already been recognized as a potential soil amendment for improving soil physicochemical properties, it could significantly improve the mutual adsorption and aggregation between soil particles after being applied to soil^34^. The maturing agent of ferrous sulfate could effectively improve soil structure and reduce the pH value of soil while loosening the soil^35^. The cementing substances such as polysaccharide and humus generated by the decomposition after applying organic fertilizer to soil could further promote the aggregation of soil particles and the stacking and cementation of micro aggregates to macroaggregates, increasing the water-stable macroaggregates content and soil structural stability^36^. Therefore, the organic–inorganic coupling treatment of fly ash + organic fertilizer can effectively increase the organic matter content of soil. In addition, fly ash has well-developed specific surface area and multi-level pores, and contains abundant clay particles and oxides, therefore it can significantly promote the formation and stabilization of the aggregates in reclaimed soil. This is similar to the stability indicators of aggregates in this study that MWD and GMD values showed a significant increase under TF, TO, TMF, TMO and TFO treatments while *D* and *E*_*LT*_ values showed a significant decrease (*P* < 0.05). Among six amendment material treatments, the indicator of aggregate stability under TFO treatment is the highest, so the coupling treatment of fly ash + organic fertilizer (TFO) has a more effective effect on improving the structural stability of hollow village reclaimed soil aggregates and may be considered as an appropriate way to increase the content and structural stability of reclaimed soil aggregates. Moreover, the coupling treatment of TFO can also significantly reduce soil bulk density and increase total nitrogen and soil moisture contents compared with other treatments, which was helpful to promote the growth and yield of maize crops. The research result is similar to the present findings of Chang et al*.* and Wei et al*.*, who indicated that the combined application of organic and inorganic modified materials were more conducive to the improvement of soil quality and crop yield than the application of either amendment alone^37,38^.

Organic fertilizer and fly ash are commonly used local waste resources, and their unreasonable utilization will lead to serious resources waste and environmental problems^39^. Reasonable conversion of waste resources into useful and sustainable materials can not only increase the soil organic matter content, but also improve soil fertility, promote plant growth and contribute to the sustainable development of agriculture^31,40^. Fly ash is waste for power plants, which, however, was very useful as a low-cost amendment for soil structure and nutrient improvement^41^. Considering the increasing cost of chemical fertilizers, Ren et al*.* and Singh et al*.* found that, within a certain range, the combined application of organic and inorganic materials could better improve the reclaimed soil structure and fertility with less cost, which was easily accepted by farmers^32,42^. Therefore, the combined treatment of organic fertilizer and fly ash (TFO) can not only meet the investment requirements in land reclamation, but also effectively improve soil fertility, and has a grear potential for application and promotion in land reclamation^43^. However, the application of inorganic modified materials alone was not conducive to the adjustment and restoration of reclaimed soil physical structure or the correction of soil nutrient imbalance, which affected the improvement of soil organic matter and microbial activity^22^. In conclusion, the preference that we recommend for the application of modified materials is as follows: fly ash + organic fertilizer (TFO) > maturing agent + organic fertilizer (TMO) > organic fertilizer (TO) > maturing agent + fly ash (TMF) > fly ash (TF) > maturing agent (TM).

### Correlation analysis between the organic matter and other parameters for the reclaimed soil in hollow village after the application of different improved materials

The application of different improved materials is the important source for the enhancement of organic matter and total nitrogen in reclaimed soil. Soil improvement materials such as organic fertilizers, maturing agents (ferrous sulfate) and fly ash, when applied alone or returned to the field after coupling, can enhance the fertility of the soil, promote the increase of crop biomass, and increase the amount of plant residues and roots returned into the soil, thus increasing the soil organic matter content^31^. In this study, after the application of different improved materials, the organic matter and total nitrogen contents in both 0–0.15 m and 0.15–0.30 m layers of the reclaimed soil showed a trend of overall increase. In the 0–0.15 m layer, the organic matter content increased by 9.6%, 79.0%, 90.0%, 61.4%, 120.1% and 131.7% respectively under TM, TF, TO, TMF, TMO, and TFO treatments compared with CK treatment, indicating that different improved materials all played important roles in improving the organic matter content of reclaimed soil. However, the improvement effect on the organic matter and total nitrogen contents of reclaimed soil differed due to the different physicochemical properties of different improved materials and the difference in the process of influencing the microbial activity in reclaimed soil. In particular, fly ash + organic fertilizer (TFO) had the best improvement effect, followed by maturing agent + organic fertilizer (TMO) and organic fertilizer (TO), which was directly related to the nutrient content and structural condition of fly ash and organic fertilizer. Fly ash is beneficial to soil microbial activity and nutrient decomposition, and endows reclaimed soil with the property of good fertilizer retention^19^. In addition, organic fertilizers are rich in organic substances and various nutrients, so the organic–inorganic coupling of fly ash + organic fertilizer (TFO) has the most significant effect on the improvement of organic matter. The research of Zhang Xuhui et al. and Six et al.^44,45^ showed that the accumulation of organic matter is closely related to 0.25–2 mm aggregates content, and the larger the particle size, the higher the organic carbon content. TFO treatment significantly increased 0.25–2 mm aggregates content, which also had an important contribution to the improvement of soil organic matter content. This was similar to the findings of Lei et al.^31,42^ which showed that the compound returning of the organic fertilizer of rotted chicken manure and the inorganic improved material of fly ash could significantly increase organic matter content.

The results of the correlation analysis between the parameters of hollow village reclaimed soil showed that the soil organic content had a highly significant positive correlation with MWD, GMD, and water-stable aggregates (particle size > 2 mm) and a highly significant negative correlation with *E*_*LT*_, *D* and micro water-stable aggregates content (particle size < 0.25 mm). The proportion of water-stable aggregates (particle size > 2 mm, 1–2 mm, and 0.5–1 mm) was significantly correlated with the values of MWD and GMD, indicators of aggregate stability, indicating that soil organic matter content affected the cementation, aggregation and structural stability of soil aggregates^46,47^. The returning and application of exogenous improved materials promoted the increase of organic matter content in hollow village reclaimed soil, which created good conditions for the cementation and aggregation of small and medium-sized soil particles and helped micro water-stable aggregates form into macro water-stable aggregates through the adhesion and aggregation of organic matter, thus enhancing the structural stability and erosion resistance of soil.

## Conclusion

After the application of different improved materials, the soil organic matter and total nitrogen contents in hollow village reclaimed soil showed an increasing trend, among which the coupling treatment of fly ash+organic fertilizer (TFO) had the best effect on the enhancement of the organic matter and total nitrogen contents in the 0–0.15 m soil layer, with an increase of 131.7% and 51.02% respectively. Meanwhile, the various improved materials significantly increased soil moisture content and reduced soil bulk density. With the increase of organic content, different amendment materials promoted the cementation and aggregation of water-stable microaggregates to water-stable macroaggregates (>0.25 mm). The water-stable macroaggregates content in the 0–0.15 m layer under different treatments showed as follows: TFO>TMO>TO>TMF>TF> TM>CK, and the largest increase was shown under fly ash+organic fertilizer treatment (TFO), being 328.2%. The values of MWD, GMD, *D* and *E*_*LT*_, indicators of aggregate structural stability, showed an overall increase, and the content and structural stability of water-stable macroaggregates in reclaimed soil were enhanced. Among the six different improved material treatments, the organic-inorganic coupling treatment of fly ash+organic fertilizer (TFO) was the most suitable way for the improvement of hollow village reclaimed soil, and it can effectively increase soil organic matter, total nitrogen, soil moisture and water-stable macroaggregates contents and enhance the structural stability and erosion resistance of the hollow village reclaimed soil, thereby improving the soil fertility.

## Materials and methods

### Experimental site

**Figure 5:**
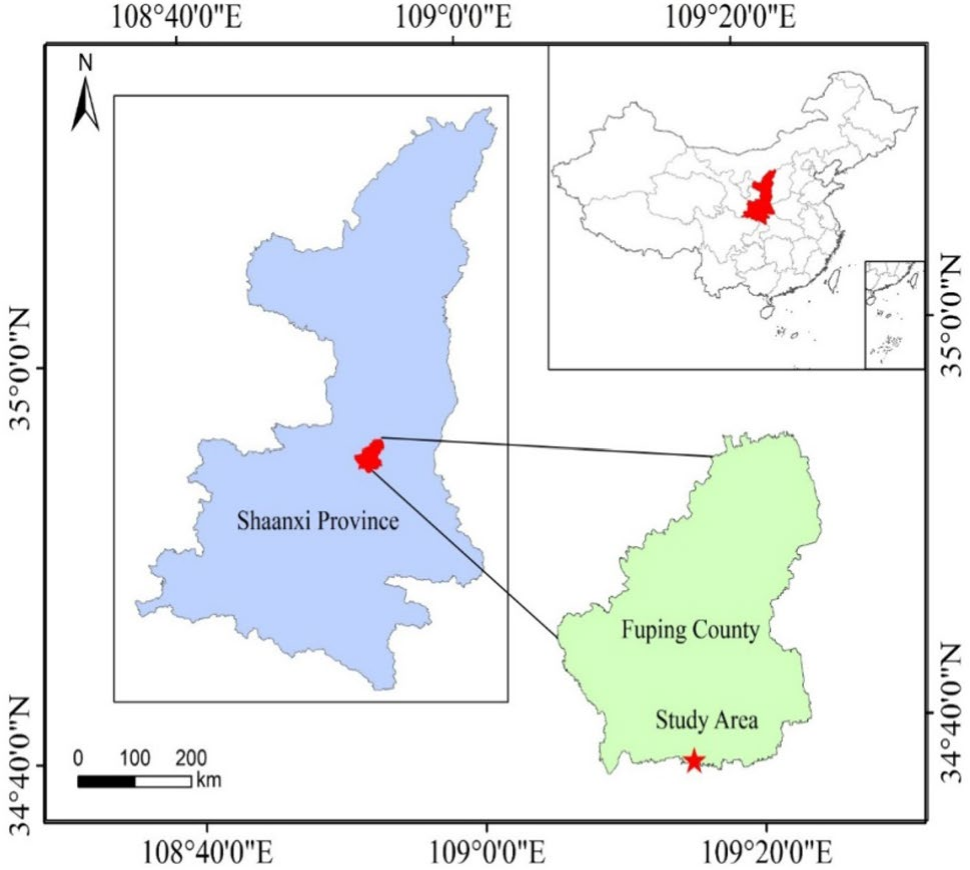
Location map of long-term field experiment area. The map was produced with ESRI ArcGIS software (version 10.3; http://www.esri.com/sofware/arcgis/arcgis-for-desktop).

**Figure 6:**
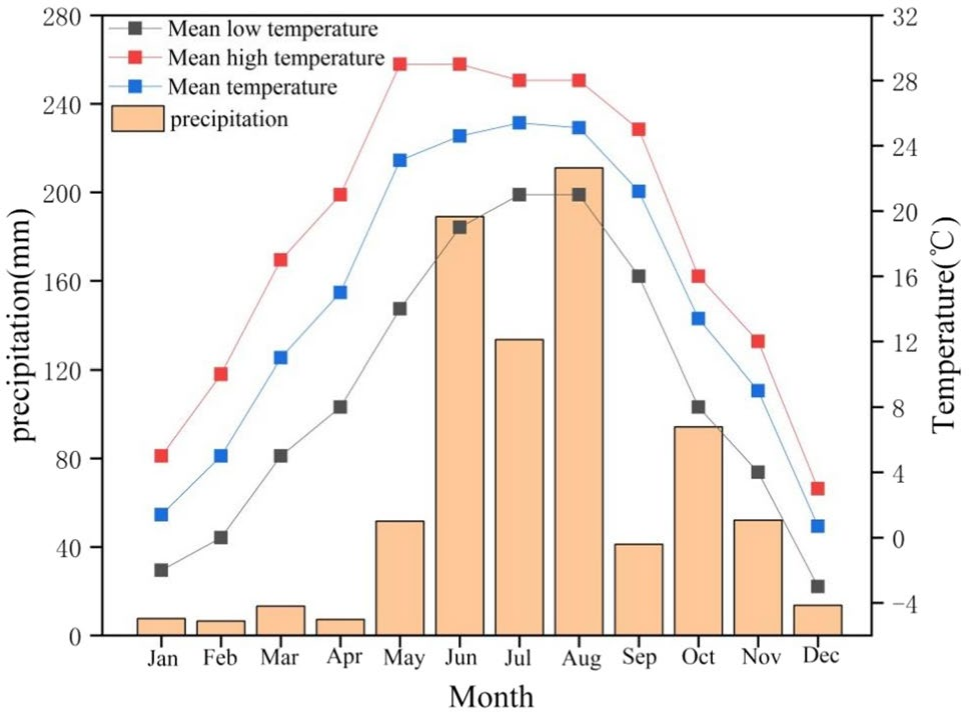
Precipitation and temperature at the experimental site in fuping county during the maize growing season in 2020.

Based on the Key Laboratory of Degraded and Unused Land Remediation Project, Ministry of Natural Resources, the long-term positioning test plot for reclaimed soil improvement of hollow villages was set up in the pilot base of Fuping County, Shaanxi Province, China (34°42′N, 109°12′E) (Fig. 5). The study area was in the Weibei loess plateau region, and the test plot was set up on June 15, 2015. The climate in this area is characterized by a continental temperate, semi-arid and semi humid one. The annual average temperature, annual average rainfall and average sunshine hours are 13.4 °C, 533.2 mm and 2,389.6 h, respectively. The precipitation and mean temperature during the maize growing season from early June to the end of September was 574.6 mm and 24.1 °C in 2020, respectively (Fig. 6). The reclamation method is to reclaim and re-organize the old wall soil of the abandoned and idle housing land through engineering techniques such as house demolition, digging and filling, land leveling and organic reconstruction of the tillage layer. In order to simulate the condition of reclaimed soil remediation and returning of abandoned housing land in hollow villages, the old wall soil of the reclaimed land remediation project of hollow villages in Chengcheng County was used for off-site backfilling, with the backfilling depth of 0.30 m. The 0.30 m below the soil layer was undisturbed soil. After removal of impurities such as rubbles and stones in the old wall soil reclaimed from the housing land, the soil was matured and structurally improved by soil fertilization and adding improved materials, so as to meet the requirements for regular growth of wheat, corn and other crops.

The basic physicochemical indicators of the reclaimed soil at the depth of 0 to 0.30 m before the the experiment were as follows: the pH value (water-soil mass ratio 1:2.5 ) was 8.5, the soil bulk density was 1.40 g cm^−3^, the soil texture was silt loam (US Soil Taxonomy) with 10.15% clay (particle size < 0.002 mm), 77.82% silt (particle size of 0.05–0.002 mm), 12.65% sand (0.05–2 mm), 4.4 g kg^−1^ organic matter, 0.15 g kg^−1^ total nitrogen, 61.3 mg kg^−1^ available potassium and 70.4 mg kg^−1^ effective phosphorus. The proportion of water-stable aggregates with particle size > 2 mm, 1 ~ 2 mm, 0.5 ~ 1 mm, 0.25 ~ 0.5 mm and < 0.25 mm are 0.67%, 0.84%, 1.67%, 3.55%, 93.27%, respectively. In summary, the reclaimed soil of the housing land in hollow villages was mainly developed from loess parent material, and the soil fertility and structure were comparatively poor.

### Experimental design

The long-term localized field improvement test of reclaimed soil in hollow villages began in June 2015. Based on the investigation and analysis of the improved materials and published literatures, fly ash, organic fertilizer (well-rotted chicken manure) and ferrous sulfate (FeSO_4_) were selected as the improved materials for reclaimed soil (Table 4). It was designed as a randomized group field test with seven treatments: maturing agent (TM), fly ash (TF), organic fertilizer (TO), maturing agent + fly ash (TMF), maturing agent + organic fertilizer (TMO), fly ash + organic fertilizer (TFO) and control (CK) treatment with no improved material added. Each treatment was repeated three times, and each plot for each treatment was a square field of 2 m × 2 m. There were a total of 21 plots for the treatments, with an isolation zone in the middle of each plot. The cropping system was a two-year triple cropping one with the rotation of winter wheat-summer maize, and the winter wheat for the test was seeded in mid-October with a seeding rate of 220.5 kg ha^−1^ and harvested in late May of the following year. And the variety was Changwu 134. The summer maize was seeded in early to mid-June with a density of 6.5 × 10^4^ plants per hectare and harvested in early October. And the variety was Xianyu 335. The maize was planted by artificial sowing, 1500 kg ha^−1^ compound fertilizer was applied before seeding, and the nitrogen, phosphorus and potassium contents in the compound fertilizer were 15%, 10% and 20%, respectively. Then, six improved materials with different treatments were evenly spread on the soil surface and mixed into the hollow village reclaimed soil at the depth of 0 to 0.30 m under artificial farming conditions, the improved materials were applied to each experimental plot once only, and other management measures and levels such as watering amount, chemical fertilizers consumption, pest control, etc. were kept consistent. See Table 1 for the specific treatment and amount of different improved materials.

**Table 4:** Experimental design of reclamation soil improvement in hollow village.

Number	Treatment	Improved Materials	Application Amount
1	CK	Control (no modified material)	0
2	TM	Maturing agent (ferrous sulfate)	0.6 t ha^−1^
3	TF	Fly ash	45 t ha^−1^
4	TO	Organic fertilizer (chicken manure)	30 t ha^−1^
5	TMF	Maturing agent + fly ash	(0.6 + 45) t ha^−1^
6	TMO	Maturing agent + organic fertilizer	(0.6 + 30) t ha^−1^
7	TFO	Fly ash + organic fertilizer	(45 + 30) t ha^−1^

### Sampling and soil physicochemical analysis

The soil samples were tested in late September 2020 after the winter wheat harvest, and the undisturbed soil samples were collected for determining soil aggregates and the mixed soil samples for determining the organic content from the soil layers at the depth of 0 to 0.15 m and 0.15 to 0.30 m respectively. Every treatment was sampled three times repeatedly. The aggregate soil samples were preserved in stainless steel aluminum boxes, and the influence on the structure of soil aggregates should be avoided as much as possible during transportation. After being brought back to the laboratory, the undisturbed soil samples were air-dried and gently broken into small pieces of about 10 mm according to the natural cracks, and impurities such as stones were removed for the testing of water-stable aggregates, organic matter and other indicators. The soil organic matter and total nitrogen contents were determined by the potassium dichromate—external heating method and Kjeldahl method^48,49^, soil bulk density and water content were determined by cutting ring method and drying method at 105 °C^50^, and the distribution and size of water-stable aggregates in two soil layers at the depth of 0–0.15 m and 0.15–0.30 m were determined by the wet-sieve method^44^. See Eqs. (1) to (4) for the indicators of aggregate structural stability such as detailed calculation of Mean Weight Diameter (MWD) and Geometric Mean Diameter (GMD), Unstable Aggregate Index (*E*_*LT*_), and Fractal Dimension (*D*)^51–53^. Maize yield was determined by random sampling method, and the maize in the 21 experimental plots with an area of about 84 square meters were manually harvested at maturity. Ten representative plants were randomly selected for each treatment at maize maturity, and the effective panicle number and number of kernels per spike were counted. The maize kernels were dried at 80 °C for 48 h to ensure that the moisture content was kept below 15%, 100-kernel weight of maize was measured by electronic balance, and the theoretical yield of maize was calculated^54^.

1 $$ {\text{MWD}} = \frac{{\sum_{i = 1}^{n} {(\overline{x}_{i} w_{i} )} }}{{\sum_{i = 1}^{n} {w_{i} } }} $$

2 $$ {\text{GMD}} = \exp \left( {\frac{{\sum_{i = 1}^{n} {w_{i} \ln \overline{x}_{i} } }}{{\sum_{i = 1}^{n} {w_{i} } }}} \right) $$

3 $$ E_{LT} = \frac{{M_{T} - R_{0.25} }}{{M_{T} }} \times 100\% $$

4 $$ \frac{{M(r < \overline{x}_{i} )}}{{M_{T} }} = \left( {\frac{{\overline{x}_{i} }}{{x_{\max } }}} \right)^{3 - D} $$

where n denotes the number of aggregate size fractions, $\overline{{x }_{i}}$ is the mean diameter of aggregates retained in the *i*th sieve, *W*_*i*_ is the weight of aggregates retained in the *i*th sieve, *M*(*r* ≤ *x*_*i*_) is the weight of aggregates with a fraction diameter less than or equal to *x*_*i*_, *M*_*T*_ is the gross weight of aggregates, and *R*_0.25_ is macroaggregates with diameters of > 0.25 mm.

### Statistical analysis

Data statistics and analysis were performed by Microsoft Excel 2010 (Microsoft, Inc., red-mond WA, USA, https://www.microsof.com/zh-cn/download/ofce.aspx) and SPSS25.0 (SPSS software,25.0, SPSS Institute Ltd, Chicago, USA, https://www.ibm.com/cn-zh/ analytics/spss-statistics-software). Differences among treatments were evaluated by one-way analysis of variance (ANOVA), the least significant difference (LSD) method was used for testing the significance of differences among different treatments (*P* < 0.05), and all data are normally distributed or close to normal distribution before ANOVA.

### Ethical statement

“Xianyu 335”, the maize (*Zea mays* L.) cultivar that we used in the present experiment, complied with institutional, national, international guidelines. We complied with the IUCN Policy Statement on Research Involving Species at risk of extinction and the Convention on International Trade in Endangered Species of Wild Fauna and Flora.

## Data Availability

All data generated or analyzed during this study are included in this published article.
